# Anti-EGFR targeted therapy delivered before versus during radiotherapy in locoregionally advanced nasopharyngeal carcinoma: a big-data, intelligence platform-based analysis

**DOI:** 10.1186/s12885-018-4268-y

**Published:** 2018-03-27

**Authors:** Hao Peng, Ling-Long Tang, Xu Liu, Lei Chen, Wen-Fei Li, Yan-Ping Mao, Yuan Zhang, Li-Zhi Liu, Li Tian, Ying Guo, Ying Sun, Jun Ma

**Affiliations:** 1 Department of Radiation Oncology, Sun Yat-sen University Cancer Center, State Key Laboratory of Oncology in Southern China, Collaborative Innovation Center for Cancer Medicine, 651 Dongfeng Road East, Guangzhou, 510060 People’s Republic of China; 2 Imaging Diagnosis and Interventional Center, Sun Yat-sen University Cancer Center, State Key Laboratory of Oncology in Southern China, Collaborative Innovation Center for Cancer Medicine, Guangzhou, People’s Republic of China; 3 Department of Clinical Trials Center, Sun Yat-sen University Cancer Center, State Key Laboratory of Oncology in Southern China, Collaborative Innovation Center for Cancer Medicine, Guangzhou, People’s Republic of China

**Keywords:** Nasopharyngeal carcinoma, Induction chemotherapy, Cetuximab, Nimotuzumab, Intensity-modulated radiotherapy, Prognosis

## Abstract

**Background:**

Little is known about the prognostic difference of anti-EGFR therapy, cetuximab (CTX) or nimotuzumab (NTZ), concurrently with induction chemotherapy (IC, investigational arm) or RT (control arm) for patients with locoregionally advanced nasopharyngeal carcinoma (LA-NPC). We conducted this retrospective study to address this.

**Methods:**

We identified 296 patients with newly diagnosed LA-NPC at Sun Yat-Sen University Cancer Center between January 2012 and May 2015. Patients were treated by IC with CCRT or RT and CTX/NTZ was delivered during IC or radiotherapy. Survival outcomes and toxicities between different arms were compared.

**Results:**

In total, there were 149 patients in the investigational arm and 147 in control arm. The 3-year disease-free survival, overall survival, distant metastasis-free survival and locoregional relapse-free survival rates for investigational arm vs. control arm were 84.3% vs. 74.3% (*P* = 0.027), 94.0% vs. 92.1% (*P* = 0.673), 88.0% vs. 81.8% (*P* = 0.147) and 93.3% vs. 88.0% (*P* = 0.093). Multivariate analysis revealed patients in the control arm achieved significantly worse disease-free survival (HR, 1.497; 95% CI, 1.016–2.206; *P* = 0.026) compared with those in the investigational arm; however, no significant difference was identified for other endpoints. Patients in the investigational arm experienced more grade 3–4 skin reaction (15.4% vs. 2.0%, *P* <  0.001) and mucositis (10.1% vs. 3.4%, *P* = 0.022) during induction phase, but less skin reaction (5.4% vs. 25.9%, *P* <  0.001) and mucositis (24.8% vs. 36.7%, *P* = 0.026) during RT.

**Conclusions:**

Our findings suggested that CTX/NTZ concurrently with IC may be a more effective and promising strategy for patients with LA-NPC receiving intensity-modulated radiotherapy.

**Electronic supplementary material:**

The online version of this article (10.1186/s12885-018-4268-y) contains supplementary material, which is available to authorized users.

## Background

Nasopharyngeal carcinoma (NPC) is a special type of head and neck malignancy for its extremely unbalanced geographic distribution and treatment modality. There are 86, 700 new cases reported worldwide in 2012 with the highest incidence in South China [1]. Unlike other head and neck cancers, radiotherapy (RT) is the primary and only cure for non-disseminated disease as a result of the anatomic constrain and sensitivity to radiation. Control of early stage disease with RT alone or chemo-radiation is usually excellent; however, management of locoregionally advanced NPC (LA-NPC) still remain unsatisfactory, with a 5-year overall survival (OS) of 67–77% [2]. Unfortunately, more than 70% of newly cases were locoregionally advanced disease at initial diagnosis [3]. Currently, concurrent chemo-radiation (CCRT) is the main standard care for LA-NPC. Although local and regional control has improved greatly, distant metastasis rates after treatment remain high and is the main source of treatment failure [4]. Therefore, identification of novel and effective therapeutic strategies is urgent and crucial for clinicians.

Epidermal growth factor receptor (EGFR), a transmembrane protein highly expressed in most human epithelial malignancies [5], is a promising therapeutic target in oncology for its correlation with aggressive phenotype, treatment resistance and poor prognosis [6, 7]. EGFR is also highly expressed in NPC [8] and numerous studies have evaluated the efficacy of anti-EGFR targeted therapy [9–15]. Cetuximab (CTX) or nimotuzumab (NTZ) (anti-EGFR monoclonal antibodies) concurrently with RT could achieved comparable outcomes compared with standard cisplatin-RT [12, 14]. When delivered during CCRT, different results were produced. You et al. and Xia et al. revealed CTX/NTZ additional to CCRT was more effective than CCRT alone [11, 13] while Li et al. did not identified any difference [10]. Regardless of the controversial efficacy, CTX/NTZ significantly increased acute mucositis and acneiform rash during RT [10, 12], resulting in poor quality of life or even disruption of RT. It seems that anti-EGFR therapy concurrent with RT may not be the best choice.

Induction chemotherapy (IC), given before RT, has been proven a promising treatment in LA-NPC for its satisfactory compliance and efficacy in reducing distant metastasis [16–19]. Possibly, CTX/NTZ in combination with IC may further reduce distant metastasis and improve survival outcomes. Notably, all abovementioned studies focus on the concurrent phase and no relative study to date has been carried out to assess the value of anti-EGFR therapy concurrently with IC. Given this concern, we initiated this retrospective study to evaluate the efficacy and toxicity difference of CTX/NTZ concurrently with IC or RT for LA-NPC.

## Methods

### Study patient

We identified 14,684 patients with newly diagnosed NPC on the big-data, intelligence database platform (YiduCloud Technology Ltd., Beijing, China) at Sun Yat-sen University Cancer center between January 2012 and May 2015. Inclusion criteria for this study were as follow: (i) stage III-IVB disease; (ii) age ≥ 18 years; (iii) karnofsky performance score (KPS) ≥ 70; (iv) did not have prior malignancies; (v) receiving IC followed by CCRT or RT alone; (vi) concurrent chemotherapy, if have, should be single-agent cisplatin; (vii) receiving intensity-modulated radiotherapy (IMRT).

### Pre-treatment staging work-up

Conventional staging workup in our center included physical examination of head and neck, direct nasopharyngoscopy, chest radiography or computed tomography (CT), magnetic resonance imaging (MRI) of head and neck, abdominal sonography, whole-body bone scan and blood profile. Positron emission tomography (PET)-CT would also be recommended for patients with advanced N (N2–3) category. Magnetic resonance (MR) or CT scans of patients were reviewed separately by two radiologists employed at our center with more than 10-year experience, and any discrepancy was resolved by consensus. Tumor stage was grouped according to the 7th edition of the International Union against Cancer/American Joint Committee on Cancer (UICC/AJCC) system [20].

### Treatment

All patients received radical IMRT at our center using the simultaneous integrated boost (SIB) technique as previously described. [18, 21] Briefly, prescribed radiation dose were 66–70 Gy at 2.12–2.27 Gy/fraction to the planning target volume (PTV) of nasopharyngeal gross tumor volume (GTV), 64–70 Gy to the PTV of GTV of metastatic lymph nodes, 60–63 Gy to the PTV of high-risk clinical target volume, and 50–56 Gy to the PTV of low-risk clinical target volume.

IC consisted of docetaxel (75 mg/m^2^ d1) with cisplatin (75 mg/m^2^ d1) (TP), fluorouracil (1000 mg/m^2^ d1-d5) with cisplatin (80 mg/m^2^ d1) (PF), or docetaxel (60 mg/m^2^ d1) plus cisplatin (60 mg/m^2^ d1) with fluorouracil (600–750 mg/m^2^ d1-d5) (TPF) every three weeks for 2–4 cycles. Concurrent chemotherapy was tri-weekly cisplatin (80–100 mg/m^2^) or weekly cisplatin (30–40 mg/m^2^).

CTX was delivered at a dose of 400 mg/m^2^ and NTZ was administered at a dose of 200 mg concurrently with IC (investigation arm) every three weeks. For patients receiving anti-EGFR therapy during RT (control arm), NTZ was administered at a dose of 200 mg weekly, and CTX was delivered at an initial dose of 400 mg/m^2^ followed by 250 mg/m^2^ weekly [13, 14]. Detailed information on treatment was presented in Additional file 1: Method S1.

### Clinical endpoints and statistical analysis

The first endpoint is disease-free survival (DFS) defined as the time from diagnosis to disease progression or death from any cause. Other endpoints included OS (time from diagnosis to death from any cause), distant metastasis-free survival (DMFS, time from diagnosis to first distant metastasis) and locoregional relapse-free survival (LRRFS, time from diagnosis to local or regional recurrence or both). Tumor response to IC was evaluated based on Response Evaluation Criteria in Solid Tumors [22]. Acute toxicities during IC were graded according to the Common Terminology Criteria for Adverse Events (version 3.0).

The Chi-square test were adopted to compare categorical variables and Mann-Whitney test for continuous variables. Survival outcomes were calculated using Kaplan-Meier method and compared by log-rank test. Multivariate cox proportional hazards model was used to estimate hazard ratios (HRs), 95% confidence intervals (CIs) and independent prognostic factors. All tests were two-sided; *P* <  0.05 was considered significant. Stata Statistical Package 12 (StataCorp LP, College Station, TX, USA) was used for all analyses.

## Results

### Patient baseline characteristics

**Fig. 1:**
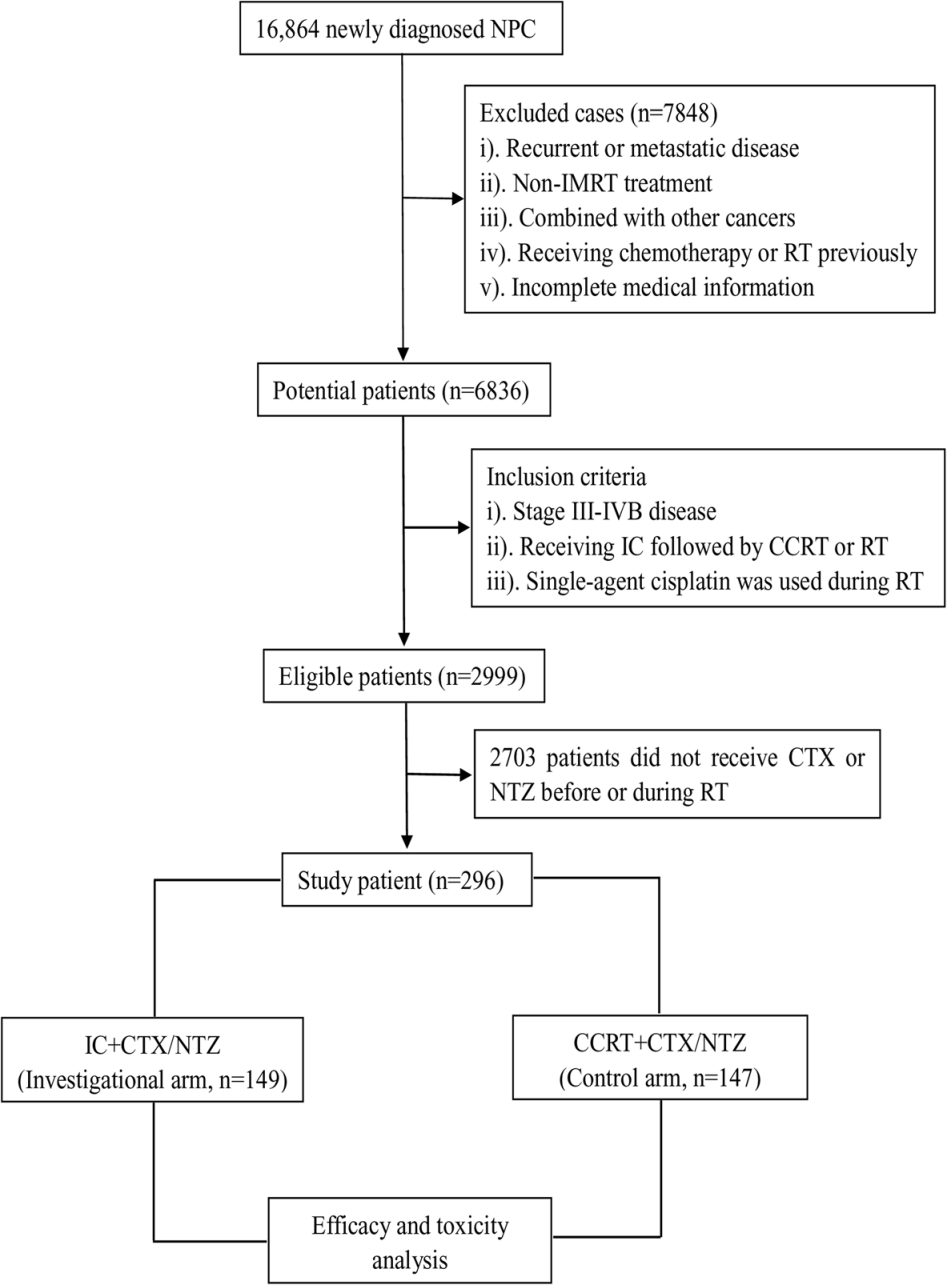
Flow chart of patient inclusion

Flow chart of patient inclusion was presented in Fig. 1. In total, we identified 2999 patients and an eventual 296 patients were eligible for this study with 149 in the investigation arm and 147 in control arm. Baseline characteristics were summarized in Table 1. The median age for the whole cohort is 42 years (range, 18–73 years), and male-to-female ratio is 3.9:1. Host, tumor and treatment related factors were well balanced between the investigational arm and control arm. Moreover, patients in these two groups had similar pre-treatment imaging stage workups (Additional file 2: Table S1).

**Table 1:** Baseline characteristics of the 296 patients with stage III-IVB nasopharyngeal carcinoma

Characteristics	Investigational arm (*N* = 149, %)	Control arm (*N* = 147, %)	*P*-value^a^
Gender			0.419
Male	116 (77.9)	120 (81.6)	
Female	33 (22.1)	27 (18.4)	
Age (years)			0.544
Median (IQR)	42 (36–51)	43 (36–52)	
Smoking			0.491
Yes	57 (38.3)	62 (42.2)	
No	92 (61.7)	85 (57.8)	
Drinking			0.095
Yes	30 (20.1)	19 (12.9)	
No	119 (79.9)	128 (87.1)	
Family history of cancer			0.668
Yes	47 (31.5)	43 (29.3)	
No	102 (68.5)	104 (70.7)	
LDH (U/L)			0.336
Median (IQR)	175 (154–216)	184 (161–215)	
Pre-DNA^b^			0.957
Median (IQR)	6880 (106–77,950)	7445 (494–52,050)	
T category^c^			0.112
T1	6 (4.0)	1 (0.7)	
T2	7 (4.7)	14 (9.5)	
T3	81 (54.4)	77 (52.4)	
T4	55 (36.9)	55 (37.4)	
N category^c^			0.873
N0	7 (4.7)	9 (6.1)	
N1	49 (32.9)	48 (32.7)	
N2	61 (40.9)	63 (42.9)	
N3	32 (21.5)	27 (18.3)	
Overall stage^c^			0.819
III	71 (47.7)	72 (49.0)	
IVA-B	78 (52.3)	75 (51.0)	
TPF regimen (cycles)			0.484
Two	15 (30.0)	14 (41.6)	
Three	33 (66.0)	18 (50.3)	
Four	2 (4.0)	2 (8.1)	
PF regimen (cycles)			0.495
Two	42 (93.3)	51 (96.3)	
Three	3 (6.7)	6 (2.9)	
Four	0 (0)	0 (0)	
TP regimen (cycles)			0.31
Two	43 (79.6)	45 (59.9)	
Three	7 (13.0)	10 (34.0)	
Four	4 (7.4)	1 (6.1)	
Concurrent chemotherapy			0.964
Yes	130 (87.2)	128 (87.1)	
No	19 (12.8)	19 (12.9)	

*IC* induction chemotherapy, *IQR* interquartile, *LDH* lactate dehydrogenase; *Pre-DNA* pre-treatment Epstein-Barr virus DNA, *TPF* docetaxel plus cisplatin with fluorouracil, *PF* cisplatin with fluorouracil, *TP* docetaxel with cisplatin

^a^*P*-values were calculated using Chi-square test for categorical variables and Mann-Whitney test for continuous variables

^b^Three patients in the control arm did not have this data

^c^According to the 7th edition of the International Union against Cancer/American Joint Committee on Cancer (UICC/AJCC) system

Among the investigational arm, 56 (37.6%) received CTX and the remaining 93 (62.4%) patients received NTZ. Within the control arm, 25 (17.0%) patients received CTX and 122 (83.0%) received NTZ. Obviously, more patients received NTZ during RT than that during IC (*P* <  0.001). Detailed information on dose and cycle of CTX/NTZ in each arm was shown in Additional file 3: Table S2. Undoubtedly, patients in the control arm received more cycles of CTX/NTZ. No dose reduction occurred in the two arms.

### Short-term efficacy after IC

Sixteen patients with N0 category were not available for regional response evaluation, with 7 (4.7%) in the investigational arm and 9 (6.1%) in the control arm. After the completion of IC, 17 (11.4%), 121 (81.2%) and 11 (7.4%) in the investigational arm, and 13 (8.8%), 118 (80.3%) and 16 (10.9%) in the control arm achieved complete response (CR), partial response (PR) and stable disease (SD), respectively (*P* = 0.476). No patient had progression disease (PD) in both arms. Additional file 4: Table S3 detailed the information on tumor response.

### Long-term outcome analysis

Up to the last visit (September 30, 2017), the median follow-up duration is 42.0 months (range 1.27–64.8). Among the whole cohort, the overall rates of locoregional and distant failures were 11.1% (33/296) and 15.9% (47/296), respectively. In detail, 26 (17.4%) in the investigational arm and 42 (28.6%) in control arm experienced treatment failure (*P* = 0.023). Additionally, 14 (9.4%) and 16 (10.9%) in the investigational and control arms died (*P* = 0.671). Three-year DFS, OS, DMFS and LRRFS rates for the whole cohort were 79.3%, 93.1%, 84.9% and 90.6%, respectively.

**Fig. 2:**
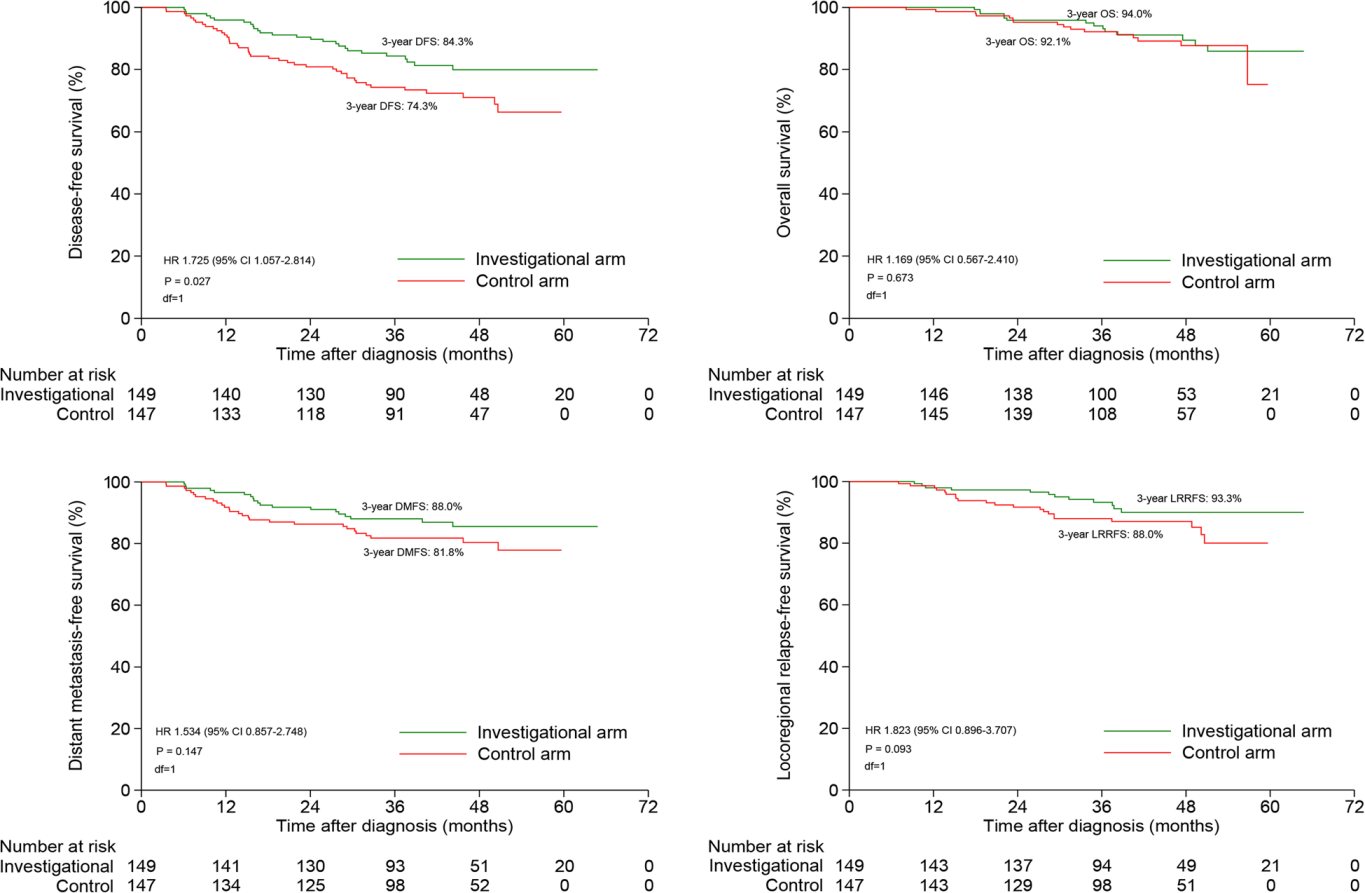
Univariate analysis comparison between the two groups

The 3-year DFS, OS, DMFS and LRRFS rates for investigational arm vs. control arm were 84.3% vs. 74.3% (*P* = 0.027), 94.0% vs. 92.1% (*P* = 0.673), 88.0% vs. 81.8% (*P* = 0.147) and 93.3% vs. 88.0% (*P* = 0.093, Fig. 2). After adjusting for various prognostic factors, patients in the control arm achieved significantly worse DFS (HR, 1.497; 95% CI, 1.016–2.206; *P* = 0.026) compared with those in the investigational arm; however, no significant difference was identified between the two arms in terms of OS (HR, 0.994; 95% CI, 0.466–2.122; *P* = 0.988), DMFS (HR, 1.409; 95% CI, 0.779–2.549; *P* = 0.251) and LRRFS (HR, 1.805; 95% CI, 0.883–3.686; *P* = 0.105; Table 2).

**Table 2:** Multivariate regression analysis for prognostic factors

Variable	HR	95% CI	*P* value^a^
Disease-free survival			
N category (N2–3 vs. N0–1)	2.512	1.388–4.548	0.002
Overall stage (IV vs. III)	1.757	1.051–2.939	0.032
Treatment group (Control vs. investigational)	1.497	1.016–2.206	0.026
Overall survival			
Overall stage (IV vs. III)	4.995	1.907–13.083	0.001
Treatment group (Control vs. investigational)	0.994	0.466–2.122	0.988
Distant metastasis-free survival			
N category (N2–3 vs. N0–1)	2.791	1.344–5.793	0.006
Treatment group (Control vs. investigational)	1.409	0.779–2.549	0.251
Locoregional relapse-free survival			
IC regimen (TPF vs. PF)	0.204	0.059–0.707	0.012
IC regimen (TPF vs. TP)	0.823	0.398–1.701	0.598
Treatment group (Control vs. investigational)	1.805	0.883–3.686	0.105

*HR* hazard ratio, *CI* confidence interval, *IC* induction chemotherapy, *TPF* docetaxel plus cisplatin with fluorouracil, *PF* cisplatin with fluorouracil, *TP* docetaxel with cisplatin, *LDH* lactate dehydrogenase

^a^Multivariate *P*-values were calculated by Cox proportional hazard regression model with backward elimination for the following prognostic factors: gender (female vs. male), age (> 42y vs. ≤ 42y), smoking (yes vs. no), drinking (yes vs. no), family history of cancer (yes vs. no), LDH (> 245 vs. ≤ 245 U/L), IC regimen (TPF vs. PF, TPF vs. TP), concurrent chemotherapy (yes vs. no), T category (T3–4 vs. T1–2), N category (N2–3 vs. N0–1), Overall stage (IV vs. III) and treatment group (control vs. investigational)

### Grade 3–4 toxicities

Acute toxicity profiles during IC and RT were evaluated between the two groups and results were presented in Table 3. Generally, grade 3–4 toxic events were comparable between the investigational and control arms (58.4% vs. 58.5%, *P* = 0.984). However, patients in the investigational arm experienced more grade 3–4 skin reaction (15.4% vs. 2.0%, *P* < 0.001) and mucositis (10.1% vs. 3.4%, *P* = 0.022) during induction phase, but less skin reaction (5.4% vs. 25.9%, *P* < 0.001) and mucositis (24.8% vs. 36.7%, *P* = 0.026) during RT compared with patients in the control arm. Haematological and gastrointestinal adverse events were similar between the two groups (all rates, *P* > 0.005).

**Table 3:** Grade 3–4 acute toxicity profiles during induction chemotherapy and radiotherapy

Grade 3–4 toxicity	Investigational arm	Control arm	*P* value^a^
	(*N* = 149, %)	(*N* = 147, %)	
Any	87 (58.4%)	86 (58.5%)	0.984
Induction phase			
Leucopenia	36 (24.2)	27 (18.4)	0.223
Neutropenia	61 (40.9)	45 (30.6)	0.064
Anaemia	2 (1.3)	2 (1.4)	0.989
Thrombocytopenia	5 (3.4)	1 (0.7)	0.102
Liver function	3 (2.0)	2 (1.4)	0.663
Renal function	0 (0)	0 (0)	1.000
Skin reaction	23 (15.4)	3 (2.0)	< 0.001
Mucositis	15 (10.1)	5 (3.4)	0.022
Nausea	3 (2.0)	2 (1.4)	0.663
Vomiting	8 (5.4)	12 (8.2)	0.338
Diarrhoea	3 (2.0)	1 (0.7)	0.321
Concurrent phase			
Leucopenia	31 (20.8)	40 (27.2)	0.197
Neutropenia	19 (12.8)	24 (16.3)	0.383
Anaemia	11 (7.4)	15 (10.2)	0.391
Thrombocytopenia	13 (8.7)	8 (5.4)	0.271
Liver function	3 (2.0)	5 (3.4)	0.462
Renal function	0 (0)	2 (1.4)	0.246
Skin reaction	8 (5.4)	38 (25.9)	< 0.001
Mucositis	37 (24.8)	54 (36.7)	0.026
Nausea	14 (9.4)	18 (12.2)	0.430
Vomiting	20 (13.4)	22 (15.0)	0.704
Diarrhoea	2 (1.3)	5 (3.4)	0.244

^a^*P*-values were calculated by Chi-square test or Fisher exact test

## Discussion

Managing advanced disease has always been a tough challenge not only in NPC management but also in many other cancers since prognosis of this subgroup is usually poor. Therefore, identification and establishment of novel and effective treatment is urgent and necessary. As far as we know, our study is the first one to compare the efficacy and safety of anti-EGFR therapy (CTX or NTZ) concurrently with IC or RT in LA-NPC treated by IMRT. We found that CTX/NTZ delivered during IC could produce significantly better DFS than administered during RT while no significant difference was achieved with regard to OS, DMFS and LRRFS.

With the wide application of IMRT in NPC, local and regional control has improved greatly and distant metastasis has become the main failure pattern [4, 23]. Although CCRT is effective, it may be not powerful enough to reduce distant metastasis for LA-NPC [24]. Additional cycles of chemotherapy like IC to CCRT is warranted. Although IC followed by CCRT has achieved excellent efficacy [16, 17, 19], further improvement of prognosis is still needed. Therefore, novel and effective treatment strategies should be identified.

EGFR on tumor cells has been established as a factor predicting treatment resistance and poor prognosis [6, 7], making anti-EGFR a potential and promising treatment strategy. Antitumor efficacy of CTX in combination with conventional chemotherapy has been proven in various EGFR-expressing malignancies like colorectal cancer, head and neck cancers and recurrent NPC [25–27]. In recurrent or metastatic head and neck squamous cell carcinoma (HNSCC), CTX combined with fluorouracil-cisplatin chemotherapy achieved significantly better DFS and OS compared with fluorouracil-cisplatin alone when given as the first-line therapy [28]. It seems that CTX could overcome resistance to previously administered chemotherapy and thereby improved survival outcomes [26]. You et al. [13] and Li et al. [10] enhanced the treatment intensity during concurrent phase by adding CTX/NTZ to standard concomitant cisplatin. However, the efficacy may be unsatisfactory and adverse events significantly increased [10, 12–14]. It’s likely that concurrent administration of anti-EGFR therapy with cisplatin is a feasible strategy but not the best. These evidence reminded us that CTX/NTZ in combination with IC may be a preferable choice. In our present study, we confirmed this view as patients in the investigational arm achieved significantly better DFS. Our findings provided a new insight in managing LA-NPC by enhancing the treatment intensity during IC.

Reasons contributing to the significantly difference of DFS were complicated. First, treatment intensity during IC was improved by adding CTX/NTZ which could help to further eradicate micro-metastasis lesions prior to RT and thereby reduce treatment failure events. Second, patients receiving CTX/NTZ during RT experienced more severe toxicities than those not. Consequently, these patients had poor quality of life during RT which could adversely affect prognosis [29, 30]. Furthermore, some patients even suffered RT interruption due to severe skin reaction or mucositis. Undoubtedly, the prolonged treatment time had a negative impact on prognosis [31, 32].

Notably, univariate and multivariate analysis only identified the significant difference between the two arms for DFS. As indicated by the survival curves, patients in the investigational arm also achieved better DMFS and LRRFS compared with patients in the control arm, although the difference was not significant. The main reason should be attributed to the small sample size which was not statistically powerful enough to identify the difference. Possibly, the improved DFS should originate from DMFS and LRRFS together. Due to the insufficient follow-up duration, no significant difference was achieved on OS although more patients in the control arm experienced treatment failure. Therefore, future studies with larger sample and longer follow-up duration are warranted to validate our results. Moreover, some other factors like program death ligand 1 (PD-L1) on tumor cells, human papillomavirus (HPV) status and lifetime cigarette smoking (pack-years) may also play important roles in prognosis and have an effect on the insignificant OS. However, these factors were not routinely evaluated in our center. Further studies were needed to evaluate these factors.

Generally, severe toxicities during RT in our study were similar as the findings in previous studies [10, 12–14]. Overall grade 3–4 toxic events were comparable between the two arms; however, anti-EGFR therapy related toxicities like skin reaction and mucositis significantly varied between the investigational and control arms during IC and RT. In detail, patients in the investigational arm experienced more grade 3–4 skin reaction and mucositis during IC but less during RT. Undoubtedly, CTX/NTZ could aggravate radiation-induced skin and oral mucositis. Another reason for the difference may be that the total dose used in induction phase is less than that used in concurrent phase. As both arms had similar chemotherapy intensity, severe heamatological and gastrointestinal events were parallel.

In our study, patients in the investigational arm had two main advantages compared with those in control arm. First, patients experienced significantly less anti-EGFR therapy related severe toxicities. Therefore, patients had better quality of life during treatment. Second, cycles of CTX/NTZ used during induction phase were usually less than that in concurrent phase. Hence, cost of anti-EGFR therapy is also less. However, limitations of this study should also be acknowledged. Our study is retrospective and the sample size may be small, meaning that potential bias existed and the power of identify difference may be insufficient. Moreover, the follow-up duration may be insufficient. Consequently, we set DFS as the first endpoint to address this. Furthermore, the cycles of IC were not uniform. In light of previous evidence, we recruited patients receiving at least two cycles because two cycles could produce the similar survival outcomes as three or more cycles [21]. Importantly, we balanced this factor between the two groups. The median age of included patients is younger than that in other reports, which should be attributed to the small sample size.

## Conclusion

In summary, CTX/NTZ concurrently with IC could achieved better survival outcomes and less severe toxicities compared with CTX/NTZ concurrently with RT in LA-NPC treated by IMRT. Our study provided a new insight into managing LA-NPC for clinicians. However, findings of present study need to be validated in prospective studies.

## Additional files

Additional file 1: Supplementary Method. (DOCX 17 kb)

Additional file 2: **Table S1.** Imaging methods for the 296 patients in the two groups. (DOCX 14 kb)

Additional file 3: **Table S2.** Cycle and total dose of CTX and NTZ for in each arm. (DOCX 13 kb)

Additional file 4: **Table S3.** Tumor response after IC between investigational and control arms. (DOCX 14 kb)

## Abbreviations

CCRT: concurrent chemo-radiation
CI: confidence interval
CT: computed tomography
CTX: Cetuximab
DFS: disease-free survival
DMFS: distant metastasis-free survival
EGFR: Epidermal growth factor receptor
GTV: gross tumor volume
HNSCC: head and neck squamous cell carcinoma
HR: hazard ratio
IC: Induction chemotherapy
IMRT: receiving intensity-modulated radiotherapy
KPS: karnofsky performance score
LA-NPC: locoregionally advanced nasopharyngeal carcinoma
LRRFS: locoregional relapse-free survival
MRI: magnetic resonance imaging
NPC: Nasopharyngeal carcinoma
NTZ: nimotuzumab
OS: overall survival
PET: positron emission tomography
PF: fluorouracil with cisplatin
PTV: planning target volume
RT: radiotherapy
SIB: simultaneous integrated boost
TP: docetaxel with cisplatin
TPF: docetaxel plus cisplatin with fluorouracil
UICC/AJCC: International Union against Cancer/American Joint Committee on Cancer
