# Supplementary material for: Anti-EGFR targeted therapy delivered before versus during radiotherapy in locoregionally advanced nasopharyngeal carcinoma: a big-data, intelligence platform-based analysis
Source: BMC Cancer. 2018 Mar 27;18:323. doi: 10.1186/s12885-018-4268-y (PMC5870169; doi:10.1186/s12885-018-4268-y)
Supplement: Supplementary file 1 — Supplementary Method. (DOCX 17 kb) [file 12885_2018_4268_MOESM1_ESM.docx]

**Additional file 1: Method S1**

***Radiotherapy***

All patients received irradiation by IMRT using the simultaneous integrated boost (SIB) technique. The target volumes were delineated according to a previously described institutional treatment protocol, which is in accordance with the International Commission on Radiation Units and Measurements reports 50 and 62. Briefly, the primary nasopharyngeal gross tumor volume (GTVnx) and cervical lymph nodes (GTVnd) were determined based on MRI/CT imaging as well as PET/CT findings. The enlarged retropharyngeal nodes together with primary gross tumor volume (GTV) were outlined as the GTVnx on the IMRT plans. The first clinical tumor volume (CTV1) was defined as the GTV within 0.5-1.0 cm margin (0.2 to 0.3 cm posterior margin) to encompass the high-risk sites of microscopic extension and the whole nasopharynx. Clinical target volume 2 (CTV2) was defined as the CTV1 plus a 0.5-1.0 cm margin (0.2 to 0.3 cm posterior margin) to encompass the low-risk sites of microscopic extension, the level of the lymph node, and the elective neck area (bilateral levels IIa, IIb, III, and Va are routinely covered for all N0 patients, whereas ipsilateral levels IV, Vb, and supraclavicular fossae were also included for the N1-3 patients). Prescribed radiation dose were 66-70 Gy at 2.12-2.27 Gy/fraction to the planning target volume (PTV) of GTVnx, 64-70 Gy to the PTV of GTVnd, 60-63 Gy to the PTV of CTV1, and 50-56 Gy to the PTV of CTV2. All patients were treated once daily with five fractions weekly. Dose constraints to the critical structures were within the tolerance according to the RTOG 0225 protocol, and efforts were made to meet the criteria as closely as possible.

***Chemotherapy***

According to the guidelines of our center and the National Comprehensive Cancer Network (NCCN), we routinely recommend radiotherapy alone for patients with stage I NPC, radiotherapy alone or CCRT for stage II disease, CCRT with or without IC for patients with stage III-IVB disease. However, concurrent chemotherapy may be eliminated after IC based on patients’ request, severe toxicities during IC and clinicians’ decisions. In our study, IC followed by CCRT or RT was delivered to all the patients. IC consisted of docetaxel (75mg/m^2^ d1) with cisplatin (75mg/m^2^ d1) (TP), fluorouracil (1000mg/m^2^ d1-d5) with cisplatin (80mg/m^2^ d1) (PF), or docetaxel (60mg/m^2^ d1) plus cisplatin (60mg/m^2^ d1) with fluorouracil (600-750 mg/m^2^ d1-d5) every three weeks for 2-4 cycles. Concurrent chemotherapy was tri-weekly cisplatin (80-100 mg/m^2^) for 2-3 weeks or weekly cisplatin (30-40 mg/m^2^) for 3-7 weeks. The cumulative cisplatin dose (CCD) during radiotherapy was calculated.

***Anti-EGFR targeted therapy***

In our center, anti-EGFR targeted therapy (Cetuximab and Nimotuzumab) was not conventionally recommended for little evidence on its efficacy and expensive cost. However, we would suggest anti-EGFR targeted therapy is patients present with N3 category or high pre-treatment plasma Epstein-Barr virus DNA which was defined as a high-risk factor of distant metastasis. CTX was delivered concurrently with IC (investigation arm) at a dose of 400 mg/m^2^ every three weeks, which was diluted in 250 mL saline and intravenously infused over 1h. Intravenous NTZ was administered at a dose of 200 mg during IC every three weeks. For patients receiving anti-EGFR therapy during RT (control arm), NTZ was administered at a dose of 200 mg weekly, and CTX was delivered at an initial dose of 400 mg/m^2^ followed by 250 mg/m^2^ weekly throughout RT.
