# Supplementary material for: Anti-EGFR targeted therapy delivered before versus during radiotherapy in locoregionally advanced nasopharyngeal carcinoma: a big-data, intelligence platform-based analysis
Source: BMC Cancer. 2018 Mar 27;18:323. doi: 10.1186/s12885-018-4268-y (PMC5870169; doi:10.1186/s12885-018-4268-y)
Supplement: Supplementary file 2 — Table S1. Imaging methods for the 296 patients in the two groups. (DOCX 14 kb) [file 12885_2018_4268_MOESM2_ESM.docx]

**Table S1**. Imaging methods for the 296 patients in the two groups.

| Staging workup | Investigational arm | Control arm | *P* value |
| --- | --- | --- | --- |
|  | (N=149, %) | (N=147, %) |  |
| **Nasopharynx and neck** | |  | 0.971 |
| MR | 78 (52.3) | 79 (53.7) |  |
| CT | 1 (0.7) | 1 (0.7) |  |
| MR + PET/CT | 70 (47.0) | 67 (45.6) |  |
| **Thorax** |  |  | 0.547 |
| Chest X-ray | 63 (42.3) | 58 (39.4) |  |
| CT | 16 (10.7) | 22 (15.0) |  |
| PET/CT | 70 (47.0) | 67 (45.6) |  |
| **Abdomen** |  |  | 0.826 |
| Abdominal ultrasound | 63 (42.3) | 59 (40.1) |  |
| CT | 15 (10.0) | 20 (13.6) |  |
| MR | 1 (0.7) | 1 (0.7) |  |
| PET/CT | 70 (47.0) | 67 (45.6) |  |
| **Bone** |  |  | 0.166 |
| ECT | 76 (51.0) | 78 (53.1) |  |
| PET/CT | 70 (47.0) | 67 (45.6) |  |
| CT | 0 (0) | 2 (1.3) |  |
| MR | 3 (2.0) | 0 (0) |  |

MR = magnetic resonance; CT = computed tomography; PET = Positron emission tomography; ECT = emission computed tomography.

^a^ *P*-values were calculated by Chi-square test or Fisher exact test.
