# Supplementary material for: Anti-EGFR targeted therapy delivered before versus during radiotherapy in locoregionally advanced nasopharyngeal carcinoma: a big-data, intelligence platform-based analysis
Source: BMC Cancer. 2018 Mar 27;18:323. doi: 10.1186/s12885-018-4268-y (PMC5870169; doi:10.1186/s12885-018-4268-y)
Supplement: Supplementary file 3 — Table S2. Cycle and total dose of CTX and NTZ for in each arm. (DOCX 13 kb) [file 12885_2018_4268_MOESM3_ESM.docx]

**Table S2**. Cycle and total dose of CTX and NTZ for in each arm.

| Cycle | Investigational arm (N = 149) | | Control arm (N = 147) | |
| --- | --- | --- | --- | --- |
|  | CTX (n=56, %) | NTZ (n=93, %) | CTX (n=25, %) | NTZ (n=122, %) |
| One | 0 (0) | 5 (5.4) | 1 (4.0) | 1 (0.8) |
| Two | 28 (50.0) | 67 (72.0) | 1 (4.0) | 2 (1.6) |
| Three | 24 (42.9) | 20 (21.5) | 1 (4.0) | 8 (6.6) |
| Four | 4 (7.1) | 1 (1.1) | 3 (12.0) | 11 (9.0) |
| Five | / | / | 9 (36.0) | 37 (30.3) |
| Six | / | / | 8 (32.0) | 56 (45.9) |
| Seven | / | / | 2 (8.0) | 7 (5.8) |

CXT = cetuximab; NTZ = nimotuzumab.
