# Supplementary material for: Anti-EGFR targeted therapy delivered before versus during radiotherapy in locoregionally advanced nasopharyngeal carcinoma: a big-data, intelligence platform-based analysis
Source: BMC Cancer. 2018 Mar 27;18:323. doi: 10.1186/s12885-018-4268-y (PMC5870169; doi:10.1186/s12885-018-4268-y)
Supplement: Supplementary file 4 — Table S3. Tumor response after IC between investigational and control arms. (DOCX 14 kb) [file 12885_2018_4268_MOESM4_ESM.docx]

**Table S3**. Tumor response after IC between investigational and control arms.

| Tumor response | Investigational arm | | Control arm | *P* value |
| --- | --- | --- | --- | --- |
|  | (N=149, %) | | (N=147, %) |  |
| **Nasopharynx** |  | |  | 0.063 |
| CR | 26 (17.4) | | 28 (19.0) |  |
| PR | 112 (75.2) | | 96 (65.3) |  |
| SD | 11 (7.4) | | 23 (15.7) |  |
| **Neck** |  | |  | 0.606 |
| CR | 35 (23.5) | | 28 (19.0) |  |
| PR | 94 (63.1) | | 92 (62.6) |  |
| SD | 13 (8.7) | | 18 (12.2) |  |
| NA (N0 category) | 7 (4.7) | | 9 (6.2) |  |
| **Nasopharynx + Neck** | |  | | 0.476 |
| CR | 17 (11.4) | | 13 (8.8) |  |
| PR | 121 (81.2) | | 118 (80.3) |  |
| SD | 11 (7.4) | | 16 (10.9) |  |

IC = induction chemotherapy; CTX = cetuximab; NTZ = nimotuzumab; CR = complete response; PR = partial response; SD = stable disease; NA = none accessible.
